# Ten Years of EWAS

**DOI:** 10.1002/advs.202100727

**Published:** 2021-08-11

**Authors:** Siyu Wei, Junxian Tao, Jing Xu, Xingyu Chen, Zhaoyang Wang, Nan Zhang, Lijiao Zuo, Zhe Jia, Haiyan Chen, Hongmei Sun, Yubo Yan, Mingming Zhang, Hongchao Lv, Fanwu Kong, Lian Duan, Ye Ma, Mingzhi Liao, Liangde Xu, Rennan Feng, Guiyou Liu, The EWAS Project, Yongshuai Jiang

**Affiliations:** ^1^ College of Bioinformatics Science and Technology Harbin Medical University Harbin 150081 China; ^2^ The EWAS Project Harbin China; ^3^ Department of Nephrology The Second Affiliated Hospital Harbin Medical University Harbin 150001 China; ^4^ The First Affiliated Hospital of Wenzhou Medical University Wenzhou 325000 China; ^5^ College of Life Sciences Northwest A&F University Yangling Shanxi 712100 China; ^6^ School of Biomedical Engineering Wenzhou Medical University Wenzhou 325035 China; ^7^ Department of Nutrition and Food Hygiene Public Health College Harbin Medical University Harbin 150081 China; ^8^ Beijing Institute for Brain Disorders Capital Medical University Beijing 100069 China

**Keywords:** epigenome‐wide association study (EWAS), epigenetics, DNA methylation

## Abstract

Epigenome‐wide association study (EWAS) has been applied to analyze DNA methylation variation in complex diseases for a decade, and epigenome as a research target has gradually become a hot topic of current studies. The DNA methylation microarrays, next‐generation, and third‐generation sequencing technologies have prepared a high‐quality platform for EWAS. Here, the progress of EWAS research is reviewed, its contributions to clinical applications, and mainly describe the achievements of four typical diseases. Finally, the challenges encountered by EWAS and make bold predictions for its future development are presented.

## Graphical abstract

Epigenome‐wide association study (EWAS) is applied to analyze DNA methylation variation in complex diseases for a decade, which is accompanied by advances and challenges. This review discusses the research process of EWAS, its application in biology and clinical translation. It also concludes with an analysis regarding the current limitations of EWAS and a forecast of its future.

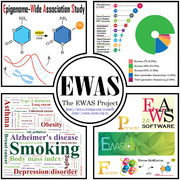

## 1 Introduction

### 1.1 Background

It has been 10 years since the concept of EWAS was introduced, and the number of EWASs on common diseases has shown an increasing trend. Similar to genome‐wide association study (GWAS), EWAS is a widely used method for identifying biomarkers in populations and discovering molecular mechanisms of disease risk.^[^ ^1^ ^]^ EWAS aims to use a variety of microarray‐based or sequencing‐based analysis techniques to obtain the association between epigenetic markers and phenotypes, which can ultimately explain the cause of the disease better and promote the development of new therapies and diagnostic methods.^[^ ^2^ ^]^

### 1.2 Rationale

**Figure 1:**
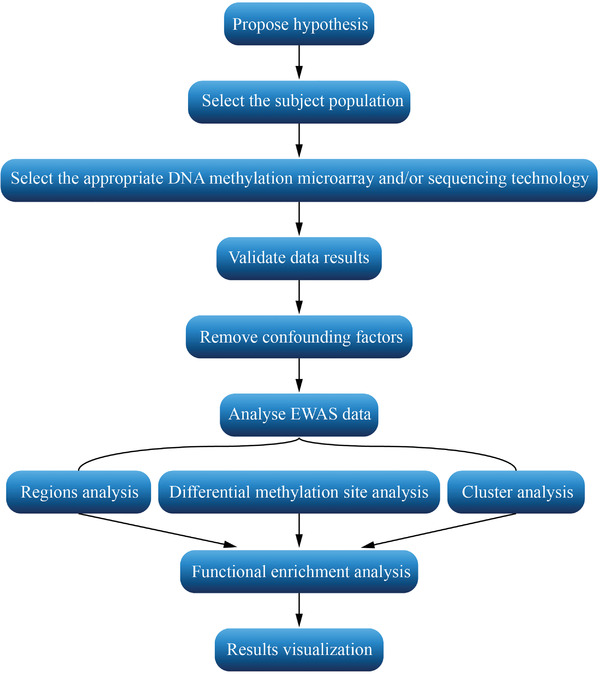
Common analysis process of EWAS.

Epigenetics is a branch of genetics, which aims to study the regulation of genes and other genetic factors in eukaryotes, covering DNA methylation, histone modification, etc.^[^ ^3^, ^4^, ^5^ ^]^ In recent years, the variation of the epigenome has become a new research direction, and the most typical epigenetic mark is DNA methylation.^[^ ^6^ ^]^ Phenotypically affected cases can be distinguished from normal samples based on the pattern of changes in DNA methylation, and this approach is known as EWAS. The commonly used EWAS analysis process usually starts with a reasonable hypothesis. Then a suitable population and tissue sample is selected. Blood samples are often used as it is difficult to obtain disease‐related tissue in most cases. However, blood DNA methylation patterns may yield different conclusions than those of tissue, so careful validation is required when using blood as a proxy. Next, it is important to choose a reasonable DNA methylation microarray or sequencing technology for factors such as experimental protocol and cost. The results are then validated and confounding factors are removed. When analyzing methylation data, it is important to focus on regional variation, identify differentially methylated regions and perform clustering analysis of CpG sites. Afterward, functional enrichment analysis will be performed to further understand the mechanisms of disease. Finally, visualization of all the analysis results is carried out in order to show them in a more intuitive way^[^ ^7^ ^]^ (**Figure** **1**).

### 1.3 Aim

The purpose of this article is to review the important findings of EWASs in biology and clinical translation. We reviewed the origin and research process of EWAS, and summarized the findings of four typical diseases. At the end of this review, we analyzed the limitations of the current experimental design and made predictions about the future of EWAS.

## 2 Research Process of EWAS

EWAS is, in particular, intriguing as a method for studying the pathogenesis of complex diseases and commonly used to analyze methylation modifications and histone modifications etc. DNA methylation is currently the most studied, which is partly due to the better chemical and temporal stability of DNA methylation and the limitations of the research tools.^[^ ^8^, ^9^, ^10^ ^]^ DNA methylation is a covalent modification with relatively good chemical stability due to the covalent binding of methyl to CpG dinucleotides.^[^ ^11^, ^12^ ^]^ From the other point of view, studies have demonstrated the temporal stability of DNA methylation.^[^ ^13^, ^14^ ^]^ In terms of the development of technical tools, there are several well established and reliable methods for the identification of DNA methylation, such as bisulfite conversion. And unlike histone modification, it is not lost during the DNA extraction process.^[^ ^15^, ^16^, ^17^ ^]^ These advantages are the reason why DNA methylation has so far been the main object of study in EWAS. In the future, as research techniques advance, other epigenetic modifications will also be widely used.

Among the methods for analyzing methylation levels, Illumina microarrays are the most widely used. It was found that in the presence of bisulfite, unmethylated cytosines of genomic DNA can be converted to uracil, while methylated cytosines remain in the cytosine state.^[^ ^18^ ^]^ Using the same principle, the Illumina Infinium HumanMethylation27 BeadChip (27k) used an Infinium I probe, while the Illumina Infinium HumanMethylation450 BeadChip (450 k) and Illumina HumanMethylationEPIC BeadChip (EPIC) used one of two probe types (Infinium I and Infinium II) to measure the methylation level of each CpG.^[^ ^19^ ^]^ Each CpG site in Infinium I is operated by two probes, one to detect “methylated (M)” intensity and the other to detect “unmethylated (U)” intensity. Infinium II uses only one probe per CpG site to distinguish methylation intensity. The methylation level of a CpG site can be expressed as a Beta value, calculated as *β* = M / (M + U + *α*), where *α* is a constant offset. For Illumina microarrays, *α* is usually given as 100.^[^ ^20^, ^21^ ^]^ Beta values range from 0 (completely unmethylated) to 1 (completely methylated), corresponding to the percentage of cells with CpG methylation.^[^ ^22^ ^]^ Beta mixture quantile normalization (BMIQ) indicates that a beta value greater than or equal to 0.75 is considered fully methylated. Beta value less than or equal to 0.25 is considered fully unmethylated. Beta value between 0.25 and 0.75 is considered hemimethylated.^[^ ^23^, ^24^ ^]^

In addition to Illumina microarrays, whole genome bisulfite sequencing (WGBS) is the most efficient method for determining the methylation status of the genome and is also the most widely used method for EWAS in next‐generation sequencing.^[^ ^25^, ^26^ ^]^ WGBS also exploits the principle that bisulfite can selectively deaminate cytosines.^[^ ^27^ ^]^ Following bisulfite treatment, polymerase chain reaction (PCR) amplification and next‐generation sequencing will be performed. Finally, untreated sequences are compared to bisulfite‐treated sequences to determine which nucleotide sites are methylated.^[^ ^28^ ^]^ Single molecule real time (SMRT) sequencing technology in the third‐generation sequencing allows direct detection of DNA methylation without the need for bisulfite conversion. In SMRT sequencing, DNA polymerase catalyses the binding of fluorescently labelled nucleotides to complementary nucleic acid strands. Information on polymerase kinetics is derived from the arrival time and duration of the resulting fluorescent pulse. Since various modifications have different effects on polymerase kinetics, the kinetic signal can be used to identify methylation levels.^[^ ^29^ ^]^

**Figure 2:**
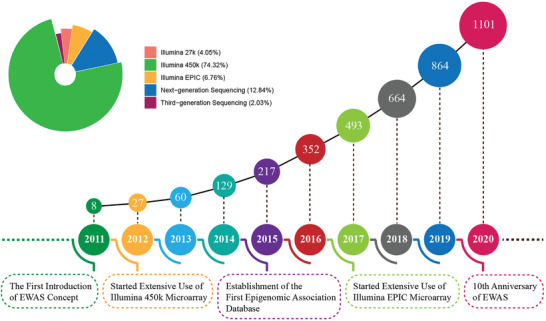
EWAS literature timeline. From 2011 to the end of 2020, the cumulative number of EWAS‐related publications per year. The pie chart shows the platforms used by EWASs in these publications.

Innovations in epigenetic methods and the reduction in the cost of EWAS have contributed to the rapid development of research. This contributes to the fact that the number of EWASs on common diseases is increasing every year (**Figure** **2**). So far, DNA methylation microarrays, next‐generation sequencing and third‐generation sequencing remain the common methods for doing high‐throughput EWAS analysis.

### 2.1 Illumina 27k

Early work on EWAS used the Illumina 27k, which contains 27 578 individual CpG loci distributed across 14 495 genes, accounting for <0.1% of the 28 million CpG loci in the human genome.^[^ ^30^ ^]^ Illumina 27k was early applied to researches on common complex diseases, the effects of drug exposure on human DNA methylation and the prediction of cancer risk.^[^ ^31^, ^32^, ^33^, ^34^ ^]^ These studies were at that time all achieving milestones and the high accuracy of Illumina 27k has been demonstrated, leading to the conclusion that Illumina 27k is feasible for most experiments. In a study shortly after the introduction of EWAS, Breitling et al. used Illumina 27k to explore differences in DNA methylation associated with smoking.^[^ ^35^ ^]^ A smoker‐specific hypermethylation site cg03636183 (*F2RL3*), which had never been identified, was found to be strongly associated with smoking‐induced disease.^[^ ^36^ ^]^ However, due to the small number of samples and controls used in experiments, replicate experiments are still required to improve the accuracy of the results. Due to the lack of coverage of CpG loci in Illumina 27k and the small experimental sample size, there are more valuable CpG loci yet to be analyzed, which will need to be confirmed in future studies.

### 2.2 Illumina 450k

As experimental techniques continue to be refined, the most widely used methylation microarray is the Illumina 450k, which contains more than 485 000 methylation sites, covering 94% of Illumina 27k microarray, as well as involves CpG sites on CpG island shores, 5'UTR, 3'UTR, first exon region, and promoter regions.^[^ ^16^, ^37^ ^]^. The Illumina 450k is the most commonly used tool for EWAS due to its wide coverage. After several years of application, researchers have recognized 1460 smoking‐related CpG sites in the EWASs with the use of the Illumina 450k.^[^ ^38^ ^]^ The CpG loci (genes) highlighted in several different studies are cg05575921 (*AHRR*), cg03636183 (*F2RL3*), and cg19859270 (*GPR15*), which have been clearly linked to a variety of inflammation‐induced diseases, such as cardiovascular diseases, metabolic diseases and cancer,^[^ ^39^, ^40^, ^41^ ^]^ There is no doubt that EWAS has made further a big stride forward with the assistance of Illumina 450k. Nevertheless, the Illumina 450k has its drawbacks. For example, its methylation site coverage is far from complete. Illumina has now released the EPIC, with new content specifically targeting regions lacking in 450k.

### 2.3 Illumina EPIC

Illumina EPIC detects the methylation status of approximately 868 564 CpG sites across the human genome, including more than 90% of the original 450K microarray (most of the loci that are not involved have been shown to perform poorly) and an additional 413 745 sites. Compared to Illumina 450k, not only does EPIC maintain comprehensive coverage of CpG islands and gene promoter regions, it also adds probe coverage of enhancer regions and gene coding regions.^[^ ^37^ ^]^ Advances in experimental techniques have made it possible to measure almost twice as many CpG sites in EPIC than in Illumina 450K and to accurately analyze the impact of DNA methylation on common diseases in a wider range of regulatory regions. In an EWAS using EPIC in 2019, six new CpG loci (genes) were revealed, including cg17739917 (*RARA*), cg14051805 (*FSIP1*), cg12956751 (*ALPP*), cg22996023 (*PIK3R5*), cg07741821 (*KIAA0087*), and cg05086879 (*MGAT3*), to be strongly associated with cancer production and progression.^[^ ^42^ ^]^ In recent years, there has been a growing number of EWASs using EPIC, which has become an indispensable tool for the study of epigenetic modifications with respect to human development and disease.^[^ ^43^, ^44^ ^]^

### 2.4 Next‐Generation Sequencing

During the past 15 years, next‐generation sequencing (NGS) has experienced high‐speed development.^[^ ^45^, ^46^ ^]^ NGS has significantly reduced sequencing costs compared to Sanger sequencing, while increasing sequencing speed and maintaining high accuracy.^[^ ^47^ ^]^ The emergence of NGS technologies has greatly influenced the development of epigenomic research and enhanced the understanding of biology and disease. One of the more widely used techniques is whole genome bisulfite sequencing (WGBS), which converts epigenetic differences into sequence differences and is used for genome‐wide DNA methylation detection.^[^ ^28^, ^48^ ^]^ The first study using WGBS to investigate Down syndrome (DS) found thousands of differentially methylated regions, with *RUNX1* being the most significant factor altering epigenetic modifications in DS.^[^ ^49^ ^]^ Although the advantages of next‐generation sequencing are huge, it is much shorter than the first‐generation sequencing technology in terms of sequence read length, which also gives room for the development of third‐generation sequencing.

### 2.5 Third‐Generation Sequencing

The advantages of third‐generation sequencing over next‐generation sequencing technology involve faster sequencing, higher accuracy, and direct detection of DNA methylation.^[^ ^50^ ^]^ In addition to its long read lengths, the PacBio SMRT sequencing technology in third‐generation sequencing, more importantly, allows the identification of methylation sites for epigenetic studies and is currently used in EWAS.^[^ ^29^, ^51^, ^52^, ^53^, ^54^ ^]^ In a recent EWAS using single‐molecule real‐time bisulfite sequencing (SMRT‐BS) to assess the impact of methylation of smoking‐associated regions on schizophrenia, allele‐specific methylation regions were identified in the smoking‐affected genes *AHRR* and *IER3*.^[^ ^55^ ^]^ Methylation CpG loci found at *AHRR* (cg05575921) and *IER3* (cg06126421) have been shown to be significantly associated with smoking, and be useful in assessing lung cancer risk in the smoking population.^[^ ^56^, ^57^, ^58^ ^]^ The results suggest that the prediction model based on DNA methylation which incorporates allele‐specific information can be applied with higher accuracy in disease research and clinical settings. As the third‐generation sequencing technology is gradually demonstrating its unique advantages, it is being used more and more in the field of epigenetics.

## 3 A Decade of EWAS Achievements

EWAS provides a systematic approach to identify epigenetic variations as biomarkers in complex diseases.^[^ ^59^, ^60^ ^]^ Biomarkers play a role in disease identification, early diagnosis, the search for drug targets and the monitoring of drug response. In recent years, it has been found that epigenomes are more likely to serve as biomarkers than transcriptomes due to their stability.^[^ ^61^ ^]^ Exploring and discovering valuable epigenetic biomarkers is gradually becoming a hot topic in disease research.^[^ ^6^ ^]^ Driven by this situation, a wide variety of EWAS‐related databases and tools have been created in response to the trend.

### 3.1 Prediction of Disease Risk

EWAS can be used to predict specific disease risk by identifying specific DNA methylation loci as biomarkers.^[^ ^62^, ^63^ ^]^ In some heritable diseases, disease‐specific biomarkers have been identified by correlating specific epigenetic traits across generations with disease.^[^ ^64^ ^]^ In this way, it is possible to determine the likelihood of the disease before it actually strikes. A study developed a methylation risk score (MRS) based on levels of methylation change. Researchers used this score together with information on 187 CpG loci associated with obesity to predict the risk of developing type 2 diabetes (T2D) in the future. The research results suggest that the MRS predicts T2D over traditional evaluation criteria like body mass index (BMI).^[^ ^65^ ^]^ Epigenetic markers are emerging as valuable predictors of human disease susceptibility, and are expected to be widely used in clinical trials in the future.

### 3.2 Early Diagnosis of Disease

Early diagnosis of the disease in a timely manner will greatly improve the outcome of the disease treatment. Indicating biomarkers early in the disease process can help alter the disease process or even stop its progression.^[^ ^66^ ^]^ Differentially methylated regions associated with autism spectrum disorders (ASD) were detected in cord blood in an EWAS.^[^ ^67^ ^]^ This study explains the progression of ASD from an epigenetic standpoint and provides new perspectives for the early diagnosis of ASD. The accurate identification of biomarkers that can be used for early diagnosis is even more important in the treatment of cancer. An EWAS has identified three CpG loci that can be used as biomarkers for the early diagnosis of colorectal cancer (CRC).^[^ ^68^ ^]^ Among them, cg04036920 and cg14472551 are located near the *KIAA1549L* transcription start site, another CpG site, cg12459502, is located in the *BCL2* body region, and all of them have high sensitivity. To date, the use of epigenetic biomarkers for early diagnosis in the clinical setting is not widespread, and biomarkers with higher accuracy are still to be discovered.

### 3.3 Identifying Drug Targets

Epigenetic drugs, as a novel therapeutic tool, are currently used mostly in cancer research. One effective way to fight cancer is to inhibit methylation, and epigenetic drugs can have an impact on DNA methylation patterns.^[^ ^69^, ^70^ ^]^ Several epigenetic drugs targeting histone methyltransferases and DNA methyltransferases are currently available for the treatment of many types of cancer.^[^ ^71^ ^]^ For instance, Zebularine, Azacitidine, and Chaetocin have been broadly used in the clinical practice.^[^ ^72^ ^]^ Epigenetic drugs are also involved in the application of neurological diseases, immunological diseases, and metabolic diseases. In an EWAS analyzing differential DNA methylation associated with childhood asthma, several genes (loci) were confirmed as drug targets, including *IL5RA* (cg01310029, cg10159529), and *KCNH2* (cg24576940, cg23147443, cg18666454).^[^ ^73^ ^]^ These targets have been widely used in a variety of drugs. *KCNH2* is the target of amiodarone hydrochloride, dofetilide, and sotalolol, *IL5RA* is the target of Benralizumab, a drug used in severe asthma patients.^[^ ^74^ ^]^ From these findings, it is clear that EWAS plays an important role in the identification of novel drug targets.

### 3.4 Measuring Drug Response by Monitoring Drug‐Induced Epigenetic Changes

Examining drug‐induced epigenetic changes is a novel way to measure drug response and evaluate prognostic ability in recent years. As epigenetic markers can offer additional perspectives on changes in biological processes, they can provide a better framework for the study of events at different stages. Longitudinal methylation studies are of great advantage in this regard.^[^ ^75^ ^]^ The main advantage is that it can explain interindividual differences in response after drug use, which is important for determining whether the drug accurately alters the response pathway associated with the disease.^[^ ^76^ ^]^ This is finally used as a criterion for drug replacement or improvement.^[^ ^77^ ^]^ In an epigenomic study on small cell lung cancer (SCLC) in 2020, the association between drug response and DNA methylation was analyzed for 526 pharmaceutical agents.^[^ ^78^ ^]^ Numerous of these drugs exhibit a strong association with *TREX1* methylation and expression. Targeting the epigenetic mechanisms of *TREX1* may be a new way to develop novel antitumor drugs.

### 3.5 The Collation of Data and Knowledge Facilitates the Researcher

As epigenetics is demonstrating a strong influence in the study of complex diseases, the new development of EWAS‐related databases provides researchers with a powerful tool. In 2013, EWASdb was released as the first database to store EWAS results, allowing researchers to look for epigenetic association results between diseases and DNA methylation.^[^ ^79^ ^]^ EWASdb contains 1319 EWASs results associated with 302 diseases/phenotypes. Furthermore, it can also search for DNA methylation markers, KEGG pathways, and GO categories that are significantly associated with certain diseases/phenotypes, which is definitely beneficial to researchers. Shortly afterward, the EWAS Atlas, a comprehensive database of EWAS knowledge, was launched.^[^ ^80^ ^]^ EWAS Atlas selects, organizes, standardizes, and presents EWAS knowledge from a wide range of publications dedicated to helping researchers understand the molecular mechanisms of epigenetic modifications. Recently, the release of the EWAS Data Hub has provided a tremendous support in resolving epigenetic mechanisms.^[^ ^81^ ^]^ It collects DNA methylation data from 75 344 samples (across 81 tissue/cell types, 6 ancestry categories, and 67 diseases) and uses efficient normalization methods to eliminate batch effects. All of these data resources have been used extensively in various EWAS experiments.

### 3.6 The Development of Tools Promotes New Discoveries

The development of practical tools related to EWAS also provides researchers with a convenient means to do so. These tools mainly perform the following functions: 1) identification of differentially methylated regions/loci (e.g., HPG‐DHunter, DMRcaller), 2) analysis of the association between epigenetic variation and disease/phenotype (e.g., EWAS2.0, EWAS1.0), 3) comprehensive analysis of DNA methylation data (e.g., GLINT, TABSAT), 4) prediction of histone modifications and DNA methylation level (e.g., Pancancer DNA Methylation Trackhub, Epigram), 5) prediction of complex traits based on methylation (e.g., TANDEM, OmicKriging), 6) identification of differential cell types based on methylation (e.g., CellDMC, BPRMeth), and 7) methylation data processing and normalization (e.g., omicsPrint, FuntooNorm). The following list (**Table** **1**) of tools is organized according to the functionality of the tool, which includes function profile, the year of release, details about the implementation, such as programming language (R, Python, Java, etc.) or web‐based browser, software availability, and PubMed ID.

**Table 1:** Summary of EWAS‐related tools

Tools	Detail	Year	Implementation	Software availability	PMID
Detection of differentially methylated region/loci					
HPG‐DHunter^[^ ^172^ ^]^	Detection of differentially methylated regions	2020	Software	https://grev‐uv.github.io/	32631226
DMRcaller^[^ ^173^ ^]^	Differentially methylated regions caller	2018	R package	http://bioconductor.org/packages/DMRcaller/	29986099
DiMmeR^[^ ^174^ ^]^	Discovery of multiple differentially methylated regions	2017	Java package	http://dimmer.compbio.sdu.dk	27794558
MethylDMV^[175]^	Detection of differentially methylated regions	2017	R package	http://www.ams.sunysb.edu/∼pfkuan/softwares.html#methylDMV	27896998
WFMM^[^ ^176^ ^]^	Identification of differentially methylated loci	2016	Software	https://biostatistics.mdanderson.org/SoftwareDownload	26559505
MethylAction^[^ ^177^ ^]^	Detection of differentially methylated regions	2016	R package	http://jeffbhasin.github.io/methylaction	26673711
AmpliMethProfiler^[^ ^178^ ^]^	Identification of methylated/unmethylated regions	2016	Python package	http://amplimethprofiler.sourceforge.net	27884103
iDNA‐Methyl^[^ ^179^ ^]^	Identification of differentially methylated loci	2015	Webserver	http://www.jci‐bioinfo.cn/iDNA‐Methyl	25596338
swDMR^[^ ^180^ ^]^	Detection of differentially methylated regions	2015	Software	http://sourceforge.net/projects/swDMR	26176536
EpiDiff^[^ ^181^ ^]^	Identification of differential epigenetic modification regions	2013	Software	http://bioinfo.hrbmu.edu.cn/epidiff	24109772
Analysis of the association between epigenetic variation and disease/phenotype					
EWAS2.0^[^ ^182^ ^]^	Analysis of the association between epigenetic variation and disease/phenotype	2018	Software	http://www.ewas.org.cn	29566144
EWAS1.0^[^ ^183^ ^]^	Analysis of the association between epigenetic variation and disease/phenotype	2016	Software	http://www.ewas.org.cn	27892496
DEMGD^[^ ^184^ ^]^	Extraction of associations of methylated genes and diseases	2013	Webserver	http://www.cbrc.kaust.edu.sa/demgd	24147091
Comprehensive Analysis of DNA Methylation Data					
^GLINT[^ ^185^ ^]^	Analysis of high‐throughput DNA‐methylation array data	2017	Python package	https://github.com/cozygene/glint/releases	28177067
TABSAT^[^ ^186^ ^]^	Analysing targeted bisulfite sequencing data	2016	Software	http://demo.platomics.com	27467908
BioVLAB‐mCpG‐SNP‐EXPRESS^[^ ^187^ ^]^	Various integrated analyses such as methylation vs. gene expression and mutation vs methylation are performed	2016	Webserver	http://biohealth.snu.ac.kr/software/biovlab_mcpg_snp_express	27477210
RefFreeDMA^[^ ^188^ ^]^	Differential DNA methylation analysis	2015	Software	http://RefFreeDMA.computational‐epigenetics.org	26673328
MethGo^[^ ^189^ ^]^	Analyzing whole‐genome bisulfite sequencing data	2015	Python package	http://paoyangchen‐laboratory.github.io/methgo	26680022
MethylSig^[^ ^190^ ^]^	DNA methylation analysis	2014	R package	http://sartorlab.ccmb.med.umich.edu/software	24836530
Methy‐pipe^[^ ^191^ ^]^	Whole genome bisulfite sequencing data analysis	2014	Software	http://sunlab.lihs.cuhk.edu.hk/methy‐pipe	24945300
RnBeads^[^ ^192^ ^]^	DNA methylation analysis	2014	Software	http://rnbeads.mpi‐inf.mpg.de	25262207
APEG^[^ ^193^ ^]^	Analyze the functions of epigenomic modifications	2013	Software	http://systemsbio.ucsd.edu/apeg	24339764
GBSA^[^ ^194^ ^]^	Analysing whole genome bisulfite sequencing data	2013	Python package	http://ctrad‐csi.nus.edu.sg/gbsa	23268441
EpiExplorer^[^ ^195^ ^]^	Analysis of large epigenomic datasets	2012	Software	http://epiexplorer.mpi‐inf.mpg.de	23034089
IMA^[196]^	Analysis of Illumina 450K	2012	R package	http://www.rforge.net/IMA	22253290
BiQ analyzer HT^[^ ^197^ ^]^	Locus‐specific analysis of DNA methylation by high‐throughput bisulfite sequencing	2011	Software	http://biq‐analyzer‐ht.bioinf.mpi‐inf.mpg.de	21565797
CNAmet^[^ ^198^ ^]^	Comprehensive analysis of high‐throughput copy number, DNA methylation and gene expression data	2011	R package	http://csbi.ltdk.helsinki.fi/CNAmet	21228048
Methyl‐analyzer^[^ ^199^ ^]^	DNA methylation analysis	2011	Python package	http://github.com/epigenomics/methylmaps	21685051
Prediction of histone modifications and DNA methylation level					
Pancancer DNA Methylation Trackhub^[^ ^200^ ^]^	Depicting the overall DNA methylation status	2018	Webserver	http://maplab.cat/tcga_450k_trackhub	29605850
LR450K^[201]^	Prediction of methylation levels	2016	R package	http://wanglab.ucsd.edu/star/LR450K	26883487
Epigram^[^ ^202^ ^]^	Predicts histone modification and DNA methylation patterns from DNA motifs	2015	Software	http://wanglab.ucsd.edu/star/epigram	25240437
MLML^[^ ^203^ ^]^	Estimates of DNA methylation and hydroxymethylation levels	2013	Software	http://smithlab.usc.edu/software/mlml	23969133
DMEAS^[^ ^204^ ^]^	Estimates methylation levels	2013	Software	http://sourceforge.net/projects/dmeas/files	23749987
Prediction of complex traits					
TANDEM^[^ ^205^ ^]^	Measure drug response	2016	R package	http://ccb.nki.nl/software/tandem	27587657
OmicKriging^[^ ^206^ ^]^	Prediction of complex traits, such as disease risk or drug response	2014	R package	http://www.scandb.org/newinterface/tools/OmicKriging.html	24799323
ITFoM^[^ ^207^ ^]^	Prediction of health risks, progression of diseases, and selection and efficacy of treatments	2013	Webserver	http://www.itfom.eu	23165094
Identification of differential cell types					
BPRMeth^[208]^	Predicting gene expression levels or clustering genomic regions or cells	2018	R package	http://bioconductor.org/packages/BPRMeth	29522078
CellDMC^[^ ^209^ ^]^	Identification of differentially methylated cell types	2018	R package	https://github.com/sjczheng/EpiDISH	30504870
eFORGE^[^ ^210^ ^]^	Identifying cell type‐specific signal	2016	Webserver	http://eforge.cs.ucl.ac.uk	27851974
Methylation data processing and normalization					
OmicsPrint^[^ ^211^ ^]^	Detection of data linkage errors in multiple omics studies	2018	R package	http://bioconductor.org/packages/omicsPrint	29420690
FuntooNorm^[^ ^212^ ^]^	Normalization of DNA methylation data	2016	R package	https://github.com/GreenwoodLab/funtooNorm	26500152
Beclear^[^ ^213^ ^]^	Correction of batch effects in DNA methylation data	2016	R package	http://bioconductor.org/packages/release/bioc/html/BEclear.html	27559732
Jllumina^[^ ^214^ ^]^	Handling of 450 k and EPIC data	2016	Java package	http://dimmer.compbio.sdu.dk/download.html	28187410
SMETHILLIUM^[^ ^215^ ^]^	Spatial normalization method for Illumina infinium HumanMethylation BeadChip	2011	R package	http://bioinfo.curie.fr/projects/smethillium	21493659

## 4 Four Exemplars of EWAS Success

**Figure 3:**
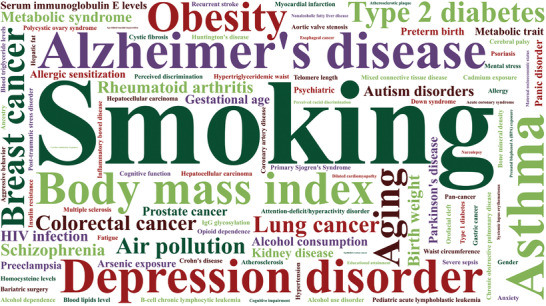
Word cloud of traits in EWASs. Top 100 traits in EWAS, including phenotypes, behaviors, environmental factors, cancer and noncancer diseases.

Published results of EWASs have addressed a variety of common diseases including autoimmune diseases (e.g., rheumatoid arthritis, asthma, and allergy), metabolic diseases (e.g., metabolic syndrome, obesity, and T2D), psychiatric disorders (e.g., alzheimer's disease, depression, and schizophrenia), and cancer (**Figure** **3**). At this point, we highlight a subcategory from each of the four broad disease categories as a typical example to illustrate some of the significant advances that were brought about by the important findings of EWASs.

### 4.1 Rheumatoid Arthritis

Epigenetics plays an important role in the pathogenesis of autoimmune diseases. In the last decade, EWAS has contributed significantly to a better understanding of the pathogenic relevance of immune‐mediated diseases. The most representative one is rheumatoid arthritis (RA), a common autoimmune disease influenced by genetic factors and environmental exposures.^[^ ^82^, ^83^ ^]^

#### 4.1.1 Biological Significance

Thousands of differential methylation sites associated with RA have been observed in nearly a decade of EWASs.^[^ ^84^, ^85^, ^86^ ^]^ The human major histocompatibility complex (MHC) has a strong epigenetic association with the pathogenesis of RA.^[^ ^87^ ^]^ A recent study detected 74 unique methylated CpG loci in the MHC region, with 22 genes containing 32 of these differentially methylated CpG motifs. These genes are involved in the antigen presentation process as well as interfering with the role of immune cells in autoimmunity.^[^ ^88^ ^]^ Another study also confirmed that differences in DNA methylation within the MHC region are strongly associated with RA progression.^[^ ^89^ ^]^ In addition to the MHC region, immune cells also have epigenetic association with RA.

Cellular and humoral immunity are the primary pathways that lead to the production of autoantibodies by immune cells that secrete inflammatory factors.^[^ ^90^ ^]^ Since RA is highly correlated with B, T, and other lymphocytes, accurate detection, and alteration of specific cell types can be achieved to effectively treat RA. Through an EWAS for RA, it was found that abnormal hypermethylation and hypomethylation of two loci, cg18972751 and cg03055671 (*CD1C* and *TNFSF10*), are associated with RA.^[^ ^91^ ^]^ The overexpression of *CD1C* in B cells enhances self‐antigen reactivity, which is one of the main causes of RA pathogenesis.^[^ ^92^ ^]^ *TNFSF10* (also known as *TRAIL*) belongs to the tumor necrosis factor (TNF) superfamily of cytokines, and it has been shown that *TRAIL* acts as a barrier against autoimmunity in RA.^[^ ^93^ ^]^ These results all clarify the causal relationship between epigenetic modifications and RA disease onset, while supporting the importance of epigenetics as a method to uncover novel molecular mechanisms in autoimmune diseases.

#### 4.1.2 Clinical Translation

DNA methylation, as an epigenetic modification affected environmental exposures covering pharmacotherapy, has been widely used in the drug discovery. Although biologic drug therapies have made tremendous advances in RA, only a minority of patients have effective control of their disease.^[^ ^94^, ^95^ ^]^ Etanercept is the most common drug used to treat RA, and an experiment characterized five drug‐sensitive methylation sites associated with it.^[^ ^96^, ^97^ ^]^ Given that some biologics are expensive and ineffective to produce, it is increasingly important to provide patients with personalized treatments and medications for RA.

### 4.2 Metabolic Syndrome

Complex diseases like metabolic syndrome (MetS) have multiple pathogenic causes, such as epigenetic mechanisms (including DNA methylation and histone modifications) along with the role of environmental factors.^[^ ^98^ ^]^ Among noncommunicable diseases, MetS has become one of the most morbid and mortal diseases.^[^ ^99^ ^]^ MetS is a combination of several diseases, covering obesity and diabetes, which significantly increases the risk of death from hepatitis, cardiovascular disease, and cancer.^[^ ^100^, ^101^, ^102^ ^]^ Due to the rapid development of EWAS in recent years, major breakthroughs have been made in the study of the etiology of MetS, factors influencing disease progression, and drug therapy.

#### 4.2.1 Biological Significance

MetS is extensively influenced by environmental factors, and diet is one of the most routine environmental factors that everyone faces in their daily lives.^[^ ^103^, ^104^ ^]^ A number of diet‐influenced methylation sites have been identified as being involved in metabolism‐related regulatory pathways, including those related to lipid metabolism, immunity, and cellular differentiation.^[^ ^105^, ^106^, ^107^ ^]^ One of the more investigated methylation sites, cg00574958, has shown significant correlation with MetS in several EWASs.^[^ ^108^, ^109^ ^]^ It was shown that carbohydrate intake leads to cg00574958 hypermethylation, which reduces the risk of MetS. Conversely, fat intake leads to cg00574958 hypomethylation, thereby increasing the risk of MetS.^[^ ^110^ ^]^

In an EWAS based on peripheral blood mononuclear cells (PBMC), methylation status of *SOCS3* (cg18181703), a gene which participates in the regulation of leptin and insulin signaling, was found to be significantly associated with obesity and was inversely proportional to BMI.^[^ ^105^ ^]^ Else, numerous studies have demonstrated that repression of *SOCS3* expression holds promise for the treatment of metabolic diseases like obesity.^[^ ^111^, ^112^, ^113^ ^]^ The contribution of epigenetics to T2D, a complex multifactorial disease, is even greater, with hundreds of methylation difference loci now identified.^[^ ^114^, ^115^, ^116^ ^]^ cg06500161 (*ABCG1*) is a well‐studied and significantly associated methylation site for T2D.^[^ ^117^ ^]^ The protein encoded by *ABCG1* is involved in intracellular as well as extracellular signaling and lipid transport, where hypermethylation of this site increases the prevalence of MetS, T2D, and obesity.^[^ ^98^ ^]^ In summary, the onset and development of MetS and related diseases (e.g., obesity and T2D) are to some extent caused by epigenetic modifications (e.g., DNA methylation).

#### 4.2.2 Clinical Translation

The EWAS on MetS provides multiple pathways for clinical translation. First, lifestyles like dietary habits alter DNA methylation patterns to varying degrees. Through several EWASs, it is clear that vitamin D, fat and alcohol intake all have impacts on MetS. Moderate alcohol and tea consumption reduces the risk of T2D and obesity, but smoking and excessive dietary fat increase the likelihood of causing T2D.^[^ ^118^, ^119^, ^120^ ^]^ Second, due to the addition of epigenetics, researchers have found promising epigenetic markers associated with T2D.^[^ ^115^, ^121^ ^]^ A study found that DNA methylation of *ABCG1* (cg06500161) affects triglyceride levels, and a drug called Pemafibrate, developed based on *ABCG1*, showed a high‐quality effect in treating T2D.^[^ ^122^, ^123^ ^]^ Third, identifying the effects of therapeutic drugs on DNA methylation based on epigenetic approaches could lead to a better understanding of the physiological pathways impacted by the drugs.^[^ ^124^ ^]^ This leads to the goal of promoting personalized therapies, developing novel diagnostic techniques and more effective medicines.^[^ ^105^ ^]^

### 4.3 Alzheimer's Disease

Alzheimer's disease (AD) is a neurodegenerative disease, the most prevalent form of dementia, affecting millions of people worldwide.^[^ ^125^ ^]^ Although the identification of psychiatric‐associated differential methylation sites started late, with the gradual maturation of EWAS, impressive results have been demonstrated in recent years in AD.^[^ ^126^, ^127^ ^]^

#### 4.3.1 Biological Significance

The potential role of epigenetic mechanisms in AD has been gradually uncovered since the first EWAS that has identified 948 CpG loci associated with AD using Illumina 27k in 2012.^[^ ^128^ ^]^ The differentially methylated sites cg11823178 and cg05066959 identified in the *ANK1* gene have been clearly pointed out in several EWASs.^[^ ^129^, ^130^, ^131^ ^]^ In addition, a large number of aberrantly methylated CpG site‐pending genes are enriched in the mitotic cell cycle regulation and Wnt signaling pathways, suggesting a hidden role for aberrant Wnt signaling in neurodegenerative diseases and promising a new drug target for AD treatment.^[^ ^132^, ^133^, ^134^ ^]^

Most EWASs for AD assess DNA methylation differences in brain tissue,^[^ ^135^, ^136^ ^]^ however, there are many sites identified in brain tissue that are not detected in blood, such as the early popular gene *ANK1*.^[^ ^66^, ^129^ ^]^ In a recent study, examining DNA methylation patterns in whole blood from AD patients, differential methylation regions were identified in gene *HOXB6*, and abnormal hypermethylation of sites within *HOXB6* (cg17179862 and cg03803541) affected granulocyte and monocyte production.^[^ ^137^ ^]^ Even more intriguingly, the same CpG sites also exhibit different or even opposite methylation patterns in the brain and blood. *OXT* (encoding oxytocin) is one of the most influential genes in the brain and blood for AD.^[^ ^138^ ^]^ In brain tissue, 10 CpG sites of *OXT* show decreased methylation levels in AD patients.^[^ ^139^ ^]^ Conversely, these sites detected elevated methylation levels in the peripheral blood. Although there are some patterns of association between the brain and blood, not all differences associated with AD in the blood are related to processes occurring in the brain, and the way in which they interact remains to be investigated.

#### 4.3.2 Clinical Translation

Epigenetics has become an important area of research in the development of drug relocalization, and the identification of protein targets based on epigenetics is now becoming the mainstream of overcoming AD.^[^ ^140^, ^141^ ^]^ Screening from known AD drugs was performed to extract 14 epigenetic drugs for relocalization based on epigenetic drug‐target network (EP‐DTN).^[^ ^142^ ^]^ There are currently no drugs that target abnormal DNA methylation loci in AD, but with the development of epigenomics and advances in pharmaceutical technology, epigenetic drugs will eventually be developed to effectively treat AD.

### 4.4 Breast Cancer

Breast cancer is the most common cancer in the female population, and its incidence is increasing every year.^[^ ^143^, ^144^ ^]^ A variety of environmental factors can contribute to the incidence of breast cancer, for instance, age, hormones, BMI, etc.^[^ ^145^ ^]^ EWAS can effectively analyze the impact of these factors on breast cancer for the purpose of supplying diagnostic and therapeutic measures.

#### 4.4.1 Biological Significance

Age is one of the risk factors for breast cancer.^[^ ^146^ ^]^ Recent EWASs have reported that some methylation sites which change with age are associated with breast cancer risk and prognosis.^[^ ^145^ ^]^ The first EWAS of age‐associated methylation changes showed that they were broadly spread throughout the genome.^[^ ^146^ ^]^ Subsequent studies have cumulatively identified over eight hundred age‐related CpG loci associated with breast cancer.^[^ ^14^, ^147^ ^]^ These results all go some way to explaining the increase in breast cancer incidence with age.

In addition to age, hormone therapy (HT) is a recognized causative factor.^[^ ^148^ ^]^ Several studies have shown that estrogen or other hormone exposure can also lead to changes in DNA methylation in the blood that can affect the risk of breast cancer.^[^ ^149^, ^150^ ^]^ An EWAS identified 694 CpG loci associated with estrogen exposure.^[^ ^151^ ^]^ Another EWAS illustrated 527 CpG sites with altered levels of DNA methylation in HT users.^[^ ^152^ ^]^ Twelve of these loci were all highly significant, such as cg01382688 (*ARHGEF4*). These findings all confirm that hormone exposure and epigenetic alterations are correlated, providing assistance in the prevention of breast cancer.

BMI may be implicated in a number of mechanisms to influence the development of breast cancer and its role should not be overlooked.^[^ ^153^ ^]^ Increased DNA methylation at cg46801642 was found to be associated with a 1.35‐fold increase in breast cancer risk in an EWAS in 2020 using blood DNA samples.^[^ ^154^ ^]^ In an EWAS of breast tissue, the methylation levels of 935 probes increased with increasing BMI and obesity was significantly associated with differential methylation in 21 CpG sites.^[^ ^155^ ^]^ These studies suggest that BMI may affect methylation levels at sites associated with breast cancer.

#### 4.4.2 Clinical Translation

Despite the remarkable success of current treatments for breast cancer, a number of patients' lives are hampered or suffer from cancer metastasis due to late detection. Extracting DNA from peripheral blood and analyzing its methylation change pattern help to find biomarkers for breast cancer risk and early detection.^[^ ^156^ ^]^ This can definitely enhance the survival rate of patients significantly. Discoveries in recent years have demonstrated the potential of epigenetic studies to assess cancer risk.^[^ ^157^ ^]^ An early study showed that reduced methylation levels of cg27091787 (*HYAL2*) were associated with an increased risk of breast cancer.^[^ ^158^ ^]^ This was followed by the generation of a large number of EWASs for the identification of DNA methylation‐related biomarkers for early detection.^[^ ^159^, ^160^ ^]^ Although dozens of biomarkers have been revealed, most of them show only very limited distinguishing power.^[^ ^157^ ^]^ Therefore, efforts are still needed to explore sensitive markers for the purpose of implementing effectively diagnostic and preventive strategies.

## 5 Discussion

We outline the development of methylation detection technologies over the last decade and then summarize the major discoveries together with associated resource tools generated by EWAS. We finally describe the biological significance and clinical translational applications of four typical diseases. In addition to these four well‐known disorders, EWAS provides strong support for the study of asthma and depression disorder in relation to methylation.

Asthma is a global disease that is influenced by environmental factors as well as epigenetic changes.^[^ ^161^ ^]^ Several EWASs have been published in recent years to investigate asthma susceptibility and mechanisms. To date, experiments using nine cohorts have yielded a total of 179 CpG loci and 36 differentially methylated regions associated with asthma.^[^ ^73^, ^162^ ^]^ Most CpG loci have strong associations with eosinophils, effector T cells, memory T cells, and natural killer cells. Currently, the main research direction for asthma‐associated EWASs is drug development, and further evaluation of methylation variants will help in the typing of asthma, which is expected to enable personalized treatment.

Depression is a common psychiatric disorder that is thought to be influenced by a combination of genetics and environment. In 2018, three methylation loci associated with depressive symptoms were identified in an EWAS that used DNA methylation in blood to identify epigenetic mechanisms of depression.^[^ ^163^ ^]^ cg04987734, cg12325605, and cg14023999 have all been associated with axon guidance pathways and may have an important role in assessing the pathology as well as the clinical role of depression.^[^ ^164^ ^]^ In an EWAS last year based on the association between DNA methylation in brain tissue and depression, reliable CpG loci were identified in the *YOD1* exon, *PFKFB2* intron, *UGT8*, *FNDC3B*, and *SLIT2* regions.^[^ ^165^ ^]^ Of these, *YOD1* has been shown to be associated with mechanisms of multiple neurodegenerative diseases and *UGT8* is a known biomarker gene for depressed mood.^[^ ^166^, ^167^ ^]^ These CpG loci hold promise as epigenetic markers of depression and for application in clinical drug development trials.

Recent researches on complex diseases, covering cancer, have been supported by EWAS due to its ability to recognize epigenetic changes that are not possible with previous technologies. EWAS is more applied to the study of the influence of environmental factors on disease mechanisms, which provides a deeper understanding of the causes and progression of diseases so that more diverse therapeutic options can be generated to achieve the goal of precision medicine.

Despite the significant achievements of EWAS, its limitations are still significant and it will face numerous challenges in the future. 1) Since epigenetic modifications are mainly influenced by environment and genetics, methylation patterns at each CpG site may be highly variable across geographic regions and races. To date, Europeans have accounted for a large proportion of the numerous EWAS subjects, although studies of Asian and African ethnicities have increased in recent years, but not nearly as much as Europeans. It has been shown that certain DNA methylation patterns vary considerably by ethnicity,^[^ ^168^, ^169^, ^170^ ^]^ which reinforces the importance of increasing the sample size and ethnic diversity of experiments, as well as to better achieve the goal of personalized treatment. 2) The number of factors that can influence epigenetic modifications is numerous, therefore high quality EWAS requires that confounding factors be considered in the design of experiments so that they can be effectively controlled in subsequent analyzes.^[^ ^61^ ^]^ Because epigenetic modifications differ between cell types and tissue types, EWAS is sometimes confounded by the cellular heterogeneity or tissue specificity of the sample. Because of the different biological characteristics of cells from different sources, whether blood samples accurately reflect the methylation patterns of the target tissues needs further validation. 3) Since methylation changes are extremely influenced by the environment, longitudinal studies are needed to analyze how epigenetic modifications change before, after the onset and following pharmacological interventions.^[^ ^171^ ^]^ Longitudinal studies allow for the detection of methylation levels in patients at multiple time points to determine the causal relationship between methylation and disease. Another advantage of longitudinal studies is the ability to access changes in epigenetic factors over the life cycle, which can help recognize biomarkers that precede the onset of disease. However, such EWAS are extremely rare because of the high costs involved and the long study duration. 4) To date, the vast majority of data sources used for EWAS are still supported by Illumina 27k or Illumina 450k, but they provide only a limited number of genomic regions, which may result in the loss of important methylation regions. The newly‐developed Illumina EPIC and three‐generation sequencing technologies have greatly improved this deficiency.^[^ ^37^ ^]^ However, the application of the new technology is not yet widespread, so future research is expected to yield more comprehensive results.

In the future, EWAS needs to find appropriate ways to address these challenges. Research also needs to move focus from methylation site discovery to biological understanding and clinical translation. For example, discovering more accurate diagnostic modalities and novel therapeutic approaches. Epigenetic changes in complex diseases will still remain a major research topic in the coming years, and for the foreseeable future the results obtained from EWAS will have a considerable impact on clinical applications.

## Conflict of Interest

The authors declare no conflict of interest.

## Author Contributions

S.Y.W., J.X.T., and J.X. contributed equally to this work, they are joint first authors. Y.S.J. conceived and contributed the work. S.Y.W., J.X.T., J.X., X.Y.C., Z.Y.W., N.Z., L.J.Z., Z.J., H.Y.C., H.M.S., Y.B.Y., M.M.Z., and H.C.L. drafted and modified the manuscript. S.Y.W., J.X.T., J.X., F.W.K., L.D., Y.M., M.Z.L., L.D.X., R.N.F., and G.Y.L. are important contributors of the EWAS Project. The EWAS Project provided data support (http://www.ewas.org.cn or http://www.bioapp.org/ewasdb).
